# Epilepsy in Leigh Syndrome With Mitochondrial DNA Mutations

**DOI:** 10.3389/fneur.2019.00496

**Published:** 2019-05-08

**Authors:** Sunho Lee, Ji-Hoon Na, Young-Mock Lee

**Affiliations:** ^1^Departments of Pediatrics, Yonsei University College of Medicine, Seoul, South Korea; ^2^Epilepsy Research Institute, Yonsei University College of Medicine, Seoul, South Korea

**Keywords:** epilepsy, mitochondria, Leigh syndrome, mitochondrial DNA, neuroimaging

## Abstract

**Background:** Leigh syndrome is a mitochondrial cytopathy that presents as a neurodegenerative disease with apparent manifestation in the central nervous system. The aim of the present study was to describe its dominant neurological clinical features and analyze data related to epilepsy in Leigh syndrome accompanied by a mitochondrial DNA mutation.

**Methods:** Whole mitochondrial sequencing was performed on 125 patients clinically suspected of Leigh syndrome. Among them, 25 patients were identified to have mitochondrial DNA associated Leigh syndrome. Electroencephalography (EEG) findings, semiology, brain imaging findings, and biochemical results, were evaluated. We also compared brain magnetic resonance imaging findings and biochemical features in patients with Leigh syndrome based on the presence of epilepsy.

**Results:** Clinical seizures were observed in 14 out of 25 enrolled patients (56%), with focal seizures being the most common type (6/14, 42.8%). All patients were found to have slow and disorganized background neural activity while eight exhibited epileptic discharges on EEG. Mutations at base pairs 10,191 and 8,993 were revealed in a relatively larger number of patients of Leigh syndrome with epilepsy. The presence of gastrointestinal symptoms was significantly more frequent in the epilepsy group (*P* = 0.042). Diffuse cerebral atrophy was significantly increased (*P* = 0.042) and cortex signal abnormalities were also increased (*P* = 0.033) in the epilepsy group.

**Conclusions:** Patients with Leigh syndrome and mitochondrial DNA mutations had a high proportion of central nervous system comorbidities, though the prevalence of epilepsy in this population was not particularly high. Various types of seizure and EEG findings are common in those with Leigh syndrome. Future imaging studies involving more patients and proper mitochondrial DNA mutation analyses are needed to further evaluate the natural course of Leigh syndrome with epilepsy.

## Introduction

Leigh syndrome is a mitochondrial disorder, the diagnosis of which is defined by three key characteristics. First, symmetrical brain stem and/or basal ganglia dysfunction on imaging is required. Patients must also exhibit delayed intellectual and motor development, and/or elevated serum or cerebrospinal fluid (CSF) lactate levels on laboratory findings, indicating abnormal energy metabolism (1).

Mitochondrial disorders are associated with heterogenous manifestations due to their involvement of multiple organs and physiological systems, though most affect the central nervous system (CNS) (2). Epilepsy is a typical phenotypic feature of mitochondrial disorders that affect the CNS (3). However, the prevalence of epilepsy is not equal among all patients with mitochondrial disorders. For instance, these rates vary among Leigh patients with West syndrome or epilepsia partialis continua (4–6), in whom the incidence of epilepsy is extremely low.

The present study aimed to describe the neurological manifestations of Leigh syndrome and, more specifically, analyze data related to epilepsy in Leigh syndrome confirmed by identification of a positive mitochondrial DNA mutation.

## Materials and Methods

### Patients and Enrollment Criteria

We performed a retrospective review of the medical records including genetic and laboratory reports of 125 patients with Leigh syndrome who had been treated at Gangnam Severance Hospital. All of the patients had confirmed mitochondrial disease, per the criteria described by Bernier et al. (7). Additionally, patients had confirmed diagnoses of Leigh syndrome (LS), as confirmed by Rahman et al. (8). These criteria include a progressive neurodegenerative disease with motor and intellectual developmental delays, signs and symptoms of brainstem and/or basal ganglia dysfunction on neuroimaging, and elevated serum or CSF fluid lactate levels. Whole mitochondrial sequencing was carried out on 125 clinically diagnosed with Leigh syndrome. Among them, 27 patients have mitochondrial DNA mutations but we excluded two patients whose mitochondrial DNA mutations were of unidentified type. Twenty five patients were genetically confirmed to have mitochondrial DNA-associated Leigh syndrome and were included in the study.

### Evaluation of Mitochondrial Disease and Leigh Syndrome

Diagnostic evaluations of patients with LS were performed. These included laboratory assessments of serum lactate levels and neuroimaging. The severity of serum lactic acidosis was defined as normal, mild (≧2-fold normal reference value), moderate (≧3-fold normal reference value). The neuroimaging modality used was brain MRI, while MR spectroscopy was used to identify a lactate peak in degenerated lesions of the brain.

Histological reports from biopsies of skeletal muscle taken from 23 patients were reviewed. Using light and/or electron microscopy, the presence of megaconia or pleoconia, or ragged red fibers were used as specific findings to indicate mitochondrial disease, while a preponderance of atrophic type I fibers were defined as non-specific findings (7). As mitochondrial chain complex (MRC) defects are defined as those below 20% of control levels (9), we also set our reduction of residual enzyme activity more strictly to <10% of controls. DNA from all patients were assessed for mitochondrial DNA (mtDNA) mutations based on a molecular sequencing analysis.

### Epilepsy Related Analysis

Factors related to epilepsy, including seizure semiology and the diagnostic evaluation tool developed by the International League Against Epilepsy (ILAE) (10) classification as well as EEG findings. Additional assessments of participant treatment responses were based on numbers of anti-epileptic drugs and seizure frequency.

### Subgrouping and Statistical Analyses

Patients were divided into two groups based on their epilepsy status. All analyses were performed using SPSS version 21.0 (IBM Corp., Armonk, NY, USA). Descriptive statistics include medians and ranges. Chi-squared distribution, the Mann-Whitney *U*-test, and the Pearson exact test were used to evaluate differences between groups. A *P* < 0.05 was considered to indicate statistical significance.

## Results

### Clinical Characteristics

In total, 25 patients were enrolled in the present study, including 10 males and 15 females. A family history of mitochondrial disease was noted in 4 of the 25 patients. The most common first onset symptom was developmental delay (40.0%), followed by seizures (12.0%). The mean age of first symptom onset was approximately 1 year (range: 0.15–9.88 years) and all patients were diagnosed within 9 years of first symptom onset. The mean duration of follow-up in our institute was over 5 years. All patients were involved in neuromuscular system treatment (100%), while over half underwent ophthalmic treatment. Developmental delays were evident alongside neuromuscular symptoms in 68.0% of patients, while seizures were evident in 32.0% (Table 1).

**Table 1:** Comparison of clinical characteristics in Leigh syndrome participants with and without epilepsy.

**General characteristics**	**Total (*N* = 25)**	**Epilepsy (*N* = 14)**	**Without Epilepsy (*N* = 11)**	***P-value***
Sex (male:female)	10 (40.0%):15 (60.0%)	7 (50.0%):7 (50.0%)	3 (27.3%):8 (72.7%)	0.414
**Family history**				
Mitochondrial disease	4/25 (16.0%)	3/14 (21.4%)	1/11 (9.1%)	0.604
Other neurologic disease	3/25 (12.0%)	1/14 (7.1%)	2/11 (18.2%)	0.565
None	18/25 (72.0%)	10/14 (71.4%)	8/11 (72.7%)	0.649
Epilepsy	0/25 (0.0%)	0/14 (0.0%)	0/11 (0.0%)	–
**First onset symptoms**				
Developmental regression	10/25 (40.0%)	7/14 (50.0%)	3/11 (27.3%)	0.414
Ptosis	4/25 (16.0%)	2/14 (14.3%)	2/11 (18.2%)	0.604
Ataxia	4/25 (16.0%)	1/14 (7.1%)	3/11 (27.3%)	0.288
Respiratory difficulty	4/25 (16.0%)	1/14 (7.1%)	3/11 (27.3%)	0.288
Seizure	3/25 (12.0%)	3/14 (21.4%)	0/11 (0.0%)	0.230
Age of 1st symptoms (years)	1.44 (0.15–9.88)	1.46 (0.16–4.65)	1.26 (0.50–9.88)	0.827
Age of muscle biopsy (years)	2.02 (0.52–20.33)	1.88 (0.52–7.33)	3.90 (1.05–20.33)	0.101
Interval from 1st symptom to confirmative diagnosis of LS (months)	7.87 (0.90–136.00)	7.85 (0.90–64.17)	9.07 (1.27–136.00)	0.488
Interval for follow up (years)	5.73 (0.74–10.94)	5.9 (0.89–10.56)	4.6 (0.74–10.98)	0.477
**Additional system involvement**				
Neuromuscular	25/25 (100.0%)	14/14 (100.0%)	11/11 (100.0%)	–
Ophthalmic	15/25 (60.0%)	10/14 (71.4%)	5/11 (45.5%)	0.241
Respiratory	9/25 (36.0%)	7/14 (50.0%)	2/11 (18.2%)	0.208
Cardiologic	8/25 (32.0%)	4/14 (28.6%)	4/11 (36.4%)	0.504
Gastrointestinal	8/25 (32.0%)	7/14 (50.0%)	1/11 (9.1%)	0.042
Endocrinologic	7/25 (28.0%)	5/14 (35.7%)	2/11 (18.2%)	0.407
Renal	4/25 (16.0%)	3/14 (21.4%)	1/11 (9.1%)	0.604
Auditory	3/25 (12.0%)	3/14 (21.4%)	0/11 (0.0%)	0.230
Urologic	3/25 (12.0%)	2/14 (14.3%)	1/11 (9.1%)	0.593
Skeletal	3/25 (12.0%)	3/14 (21.4%)	0/11 (0.0%)	0.230
**Neuromuscular symptoms**				
Developmental delay	17/25(68.0%)	9/14 (64.3%)	8/11 (72.7%)	0.496
Seizure	8/25 (32.0%)	8/14 (57.1%)	0/11 (0.0%)	0.003
Dystonia	7/25 (28.0%)	5/14 (35.7%)	2/11 (18.2%)	0.407
Ataxia	5/25 (20.0%)	2/14 (14.3%)	3/11 (27.3%)	0.623
Spasticity	5/25 (20.0%)	4/14 (28.6%)	1/11 (9.1%)	0.341
Dysarthria	3/25 (12.0%)	1/14 (7.1%)	2/11 (18.2%)	0.565
Psychomotor delay	2/25 (8.0%)	0/14 (0.0%)	2/11 (18.2%)	0.183
Muscle weakness	1/25 (4.0%)	1/14 (7.1%)	0/11 (0.0%)	0.560

### Neuroimaging and Diagnostic Evaluation of Leigh Syndrome

Increased signal intensity in the basal ganglia (92.0%) and brain stem (76.0%) on MRI were both observed, with over a half (84%) of patients exhibiting a lactate peak on plasma MR spectroscopy. An additional 20 patients exhibited mildly increased serum lactate levels. Electron microscopic changes were identified, including pleoconia (34.8%) or megaconia (26.0%). Mitochondrial respiratory chain complex 4 defects were also detected in 12 patients, and complex 1 defects in 11 patients (Tables 2, 3).

**Table 2:** Comparison of neuroimaging findings in Leigh syndrome participants with and without epilepsy.

**Neuroimaging findings**	**Total (*N* = 25)**	**Epilepsy (*N* = 14)**	**Without Epilepsy (*N* = 11)**	***P-value***
**MRI ABNORMALITIES**				
Basal ganglia	23/25 (92.0%)	14/14 (100.0%)	9/11 (81.8%)	0.183
Brain stem	19/25 (76.0%)	12/14 (85.7%)	7/11 (63.6%)	0.350
Cerebellar atrophy	15/25 (60.0%)	9/14 (64.3%)	6/11 (54.5%)	0.697
Thalamus	12/25 (40.0%)	9/14 (64.3%)	3/11 (27.3%)	0.111
Diffuse cerebral atrophy	11/25 (44.0%)	9/14 (64.3%)	2/11 (18.2%)	0.042
Cortex	9/25 (30.0%)	8/14 (57.1%)	1/11 (9.1%)	0.033
White matter	7/25 (25.0%)	6/14 (42.9%)	1/11 (9.1%)	0.09
**MR SPECTROSCOPY**				
Lactate peak	15/22 (68.2%)	8/13 (61.5%)	7/9 (77.8%)	0.648

*MR, magnetic resonance; MRI, magnetic resonance imaging*.

**Table 3:** Comparison of diagnostic evaluation between Leigh syndrome participants with and without epilepsy.

**Diagnostic evaluation**	**Total**	**Epilepsy (*N* = 14)**	**Without epilepsy (*N* = 11)**	***P-value***
**LACTIC ACIDOSIS (*****N*** **=** **25)**				
Within normal range	4/25 (16.0%)	1/14 (7.1%)	3/11 (27.3%)	0.288
Mildly increased (<2-fold)	20/25 (80.0%)	12/14 (85.7%)	8/11 (72.7%)	0.623
Moderated increased (2–3-fold)	1/25 (4.0%)	1/14 (7.1%)	0/11 (0.0%)	0.560
Severe increased (≥3-fold)	0/25 (0.0%)	0/14 (0.0%)	0/11 (0.0%)	–
**MUSCLE BIOPSY OBTAINED (*****N*** **=** **23)**				
**Light microscopic changes**				
Specific findings for mitochondrial disease	4/23 (17.4%)	2/13 (15.4%)	2/10 (20.0%)	0.596
Non-specific findings	2/23 (8.7%)	1/13 (7.7%)	1/10 (10.0%)	0.692
**Electron microscopic changes**				
Pleoconia	8/23 (34.8%)	4/13 (30.4%)	4/10 (40.0%)	0.685
Megaconia	6/23 (26.0%)	4/13 (30.8%)	2/10 (20.0%)	0.660
**MRC enzyme assay**				
Complex IV defect	12/23 (52.2%)	6/13 (46.2%)	6/10 (60.0%)	0.680
Complex I defect	11/23 (47.8%)	7/13 (53.8%)	4/10 (40.0%)	0.680

*MRC, mitochondrial chain complex*.

### Mitochondrial DNA Mutations in Leigh Syndrome

We analyzed 16,500 base pairs of mitochondrial DNA using direct sequencing to confirm the most reliable mutations related to Leigh syndrome based on established genetic reports. Mutations at base pairs 10,191, 13,513, and 8,993 were revealed in relatively larger number of patients suffering Leigh syndrome with epilepsy (Table 4).

**Table 4:** Comparison of mtDNA mutations between Leigh syndrome participants with and without epilepsy.

**mtDNA mutations**	**Total (*N* = 25)**	**Epilepsy (*N* = 14)**	**Without epilepsy (*N* = 11)**	***P-value***
10191 T>C	4/25 (16.0%)	4/14 (28.6%)	0/11 (0.0%)	0.105
13513 G>A	4/25 (16.0%)	1/14 (7.1%)	3/11 (27.3%)	0.288
8993 T>G	4/25 (16.0%)	2/14 (14.3%)	2/11 (18.2%)	0.604
8993 T>C	3/25 (12.0%)	1/14 (7.1%)	2/11 (18.2%)	0.565
9176 T>C	3/25 (12.0%)	1/14 (7.1%)	2/11 (18.2%)	0.565
3697 G>A	2/25 (8.0%)	1/14 (7.1%)	1/11 (9.1%)	0.697
10158 T>C	1/25 (4.0%)	1/14 (7.1%)	0/11 (0.0%)	0.560
10744 A>G	1/25 (4.0%)	1/14 (7.1%)	0/11 (0.0%)	0.560
11777 C>A	1/25 (4.0%)	1/14 (7.1%)	0/11 (0.0%)	0.560
14459 G>A	1/25 (4.0%)	0/14 (0.0%)	1/11 (9.1%)	0.440
9185 T>C	1/25 (4.0%)	0/14 (0.0%)	1/11 (9.1%)	0.440

### Epilepsy in Leigh Syndrome

A total of 14 patients with Leigh syndrome in the present study were confirmed to also have epilepsy (Table 5). The mean age of seizure onset among these individuals was approximately 2 years. During the initial stage, their seizure frequency reported was about seven times per month. We found that six patients (42.8%) had a focal seizure locus while 5 (35.7%) had generalized seizure semiology. Complex partial seizure was the most common (3/14) (21.4%) type of focal seizure observed. As for generalized seizure types, myoclonic seizure, tonic seizure, atonic seizure, and tonic-clinic seizure were observed with relatively even prevalence among patients. In addition, two patients showed partial seizure with secondary generalization and one patient had unclassified epileptic seizures.

**Table 5:** Characteristics of epilepsy in Leigh syndrome patients (total *N* = 14).

Age of first seizure (years)	2.10 (0.12–4.92)^*^
Initial seizure frequency/month	7.14 (1–24)^*^
**SEIZURE TYPE**	
Focal seizures	6/14 (42.8%)
Simple partial without motor signs	1/14 (7.1%)
Simple partial with motor signs	2/14 (14.3%)
Complex partial	3/14 (21.4%)
Generalized seizures	5/14 (35.7%)
Myoclonic seizures	2/14 (14.3%)
Tonic seizures	1/14 (7.1%)
Atonic seizures	1/14 (7.1%)
Tonic-clonic seizures	1/14 (7.1%)
Partial seizure with secondary generalization	2/14 (14.3%)
Unclassified epileptic seizures	1/14 (7.1%)
Mixed type of seizures	4/14(28.6%)
**ELECTROENCEPHALOGRAPHY**	
**Background rhythm**	
Slow and disorganized	14/14 (100.0%)
Asymmetric focal slowing	6/14 (42.8%)
**Epileptiform discharges**	
No epileptic discharges	6/14 (42.8%)
Focal sharp/spike waves	5/14 (35.7%)
Multifocal sharp/spike waves	1/14 (7.1%)
Generalized sharp and waves	2/14 (14.3%)
Generalized paroxysmal fast activities	2/14 (14.3%)
**Electrographic seizure**	
Generalized seizure	1/14 (7.1%)
Focal seizure	2/14 (14.3%)
Numbers of status epilepticus	0/14 (0.0%)
**TREATMENT OPTIONS**	
With antiepileptic drugs (AEDs)	12/14 (85.7%)
Levetiracetam	4/14 (28.6%)
Lamotrigine	3/14 (21.4%)
Oxecarbamazepine	3/14 (21.4%)
Zonisamide	3/14 (21.4%)
Topiramate	2/14 (14.3%)
Phenytoin	1/14 (7.1%)
Carbamazepine	1/14 (7.1%)
Numbers of initial treatment	0.7 (0–3.0)^*^
No AED	2/14 (14.3%)
1 AED	8/14 (57.1%)
2 AEDs	3/14 (21.4%)
3 AEDs	1/14 (7.1%)
Numbers of last visit	1.6 (0–5.0) ^*^
No AED	2/14 (14.3%)
1 AED	6/14 (42.9%)
2 AEDs	2/14 (14.3%)
3 AEDs	2/14 (14.3%)
>3 AEDs	2/14 (14.3%)
Ketogenic diet	2/14 (14.3%)
**RESPONSE TO AEDs AFTER 1 YEAR**	
Seizure free	6/14 (42.8%)
Decreased seizure frequency	3/14 (21.4%)
>90% reduction of seizures frequency	1/14 (7.1%)
50–90% reduction of seizures frequency	1/14 (7.1%)
<50% reduction of seizures frequency	1/14 (7.1%)
No change of seizure frequency	2/14 (14.3%)
Increased seizure frequency	1/14 (7.1%)
Seizure free at last visit	8/14 (57.1%)

* *Marked the median with interquartile range*.

All patients showed slow and disorganized background neural activity while eight exhibited epileptic discharges on EEG. Among them, five patients had focal sharp or spike waves, accounting for 35.7% of the total. In this study, none of the patients with Leigh syndrome experiencing epilepsy had an episode of status epilepticus.

A total of 12 patients (85.7%) were taking anti-epileptic drugs at the time. Eight patients were taking only one AED (57.1%). However, four patients failed to control their seizure within 1 year of their initial treatment and went on to taking two or more types of AEDs. During an average follow-up period of 5.7 years, eight patients were taking one AED at the time of the last visit, accounting for 57.1% of the total. However, the number of patients taking three or more multiple AEDs was four (28.6%). Two patients were on the ketogenic diet and did not fully respond to multiple AEDs.

After we started the AED, we summarized the treatment effects of the patients. Six patients became seizure free and three patients achieved decreased seizure frequency. There were two patients with no change of seizure frequency and one patient suffered increased seizure frequency. However, over the entire follow-up period, seizure free status was observed in 8 out of 14 patients.

### Comparison of Those With Leigh Syndrome With and Without Comorbid Epilepsy

We divided our participants, all of whom had Leigh syndrome, into two groups based on their epilepsy status groups and presence of mtDNA mutations. We found no statistically significant differences in the clinical characteristics of the two groups, except for their gastrointestinal system symptoms and seizure history (Table 1). The presence of gastrointestinal symptoms was significantly higher in the epilepsy group (50.0 vs. 9.1%, *P* = 0.042).

A comparison of MRI findings between the two groups revealed that diffuse cerebral atrophy was significantly increased with epilepsy (64.3 vs. 18.2%, *P* = 0.042). Cortex signal abnormalities with epilepsy were also increased (57.1 vs. 9.1%, *P* = 0.033). Abnormal signals in other areas on MRI and lactate peaks on MR spectroscopy did not differ between the epilepsy and non-epilepsy groups. Additionally, there were no statistically significant differences in lactic acidosis, muscle biopsy findings, mitochondrial chain complex defects, or mitochondrial DNA mutation types between the two groups.

## Discussion

Epilepsy is one of the most significant clinical manifestations of mitochondrial disorders. Mitochondrial chain impairments are often caused by neuronal mitochondrial DNA damage, which is an aspect of epileptogenesis (11). The incidence of epilepsy in mitochondrial encephalopathy presents ranges widely, though most studies report 35–61% (11–13). In our study, 14 out of 25 patients had epilepsy, which corresponds to a morbidity of 56%. This was within the range of the existing reference but on the higher side. The prevalence of epilepsy in individuals with Leigh syndrome ranges sporadically, but is generally high, similar to that of mitochondrial disorder in individuals with epilepsy (3, 5, 14). Other mitochondrial disorders accompanying epilepsy, such as Mitochondrial encephalopathy, lactic acidosis, and stroke-like episodes (MELAS) and Myoclonic epilepsy with ragged red fibers (MERRF), were reported by Lee et al. to have a similarly high prevalence (4, 15). Despite this, seizure is not included as a diagnostic feature of Leigh syndrome. However, epilepsies in individuals with Leigh syndrome are often noted to be relatively high, possibly due to the confusion of seizures with other neuromuscular symptoms such as dystonia or spasticity. The present study indicates that the incidence of epilepsy in Leigh syndrome patients falls within previously established ranges. Given this, we conclude that the incidence of epilepsy may differ from that of subtypes of mitochondrial disorders, as there is cortical dysfunction in each which may limit the accuracy of a purely syndromic diagnosis.

Cortical abnormalities revealed via neuroimaging (e.g., MRI) are often used to indicate epilepsy (16, 17). Similarly, ubiquinone deficiencies, MELAS, POLG (polymerase subunit gamma) mutation, and Kearns-Sayre Syndrome are all associated with various lesions and brain MRI abnormalities, including alterations in the cerebral cortex, seizure, or stroke-like episodes (18). However, most Leigh patients also exhibit abnormalities in the brain stem or basal ganglia, with rare cases exhibiting cortical lesion on MRI (19, 20). In the present study, the proportion of patients with Leigh syndrome revealed to have some cortical abnormalities on MRI was over half, especially in those with epilepsy, indicating that cortical changes on neuroimaging may reflect impending epilepsy in Leigh syndrome. Mitochondrial disorders often feature critical dysfunctions in the CNS, which stem from the high-energy demands of the CNS, resulting in diffuse or focal brain tissue atrophy (9, 21). The present study reveals a relatively high proportion of cerebral atrophy among those with Leigh syndrome and significantly increased rates in those with epilepsy. It is possible that epilepsy's high metabolic cost may lead to accelerated central nervous dysfunction with mitochondrial dysfunction, ultimately leading to the deterioration that occurs in the natural course of Leigh syndrome with comorbid epilepsy. Sofou et al. found that multiple factors, including epilepsy and birth defects, resulted in exacerbated Leigh syndrome. Typically, patients with Leigh syndrome are thought to live 2 or 3 years beyond first symptom onset, with those with late onset Leigh syndrome living up to 20 years of age. However, in most patients, deterioration begins to accelerate at ~20 months of age (22, 23). In patients with Leigh syndrome with comorbid epilepsy, timely evaluation including neuroimaging is particularly critical, as their symptoms may worsen and have deteriorating prognosis. Based on the results that diffuse atrophy is more associated with Leigh syndrome with epilepsy, it can serve as a good imaging indicator of disease progression. So, we strongly suggest that neuroimaging may provide critical clues about the disease course and may be helpful for the detection of epilepsies among patients with Leigh syndrome.

Mitochondrial dysfunction leads to various, non-specific clinical phenotypes including gastrointestinal symptoms such as dysphagia, malnutrition, gastroparesis, and constipation (8). Gastrointestinal dysmotility may be caused by decreased mitochondrial proliferation and thus leads to marked atrophy of the external layer of the muscular propria (24). Some studies of mitochondrial neuro-gastro-intestinal encephalopathy (MNGIE) have also reported its association with non-specific neurologic symptoms, such as ophthalmoplegia and ptosis 9 (25, 26). In their analysis of patients with MELAS, de Laat et al. found that 20% experienced dysphagia and constipation while 40% experienced gastroparesis, which often contributes to nutrition absorption failures and malnutrition (27). Gastrointestinal symptoms may be due to either central or peripheral neuropathy, though epilepsies such as panayiotopoulos epilepsy or the aura that accompanies a focal epilepsy are often misdiagnosed as celiac disease or other gastrointestinal diseases (28) and thus the connection between these symptoms and epilepsies remains poorly understood. For instance, Yeh et al. reported a high risk of gastrointestinal hemorrhage in epilepsy patients (29), though this was not correlated with the severity of the epilepsy or considered to be a risk factor for future seizures. Similarly, in the present study, we found a relatively high proportion of gastrointestinal symptoms among patients with Leigh syndrome. This incidence of gastrointestinal symptoms was significantly increased among those with epilepsy. Gastrointestinal dysfunctions in patients with Leigh syndrome may be caused by malabsorption or malnutrition, which can lead to epileptogenesis (30). Malnutrition also exacerbates immunodeficiencies and increases infection risk via blunting cell-mediated immunity, phagocytosis, and complementary system activity, which collectively also increase risk for epilepsy. Additionally, malabsorption can result in micronutrient deficiencies of calcium, magnesium, sodium, and other vital molecules, altering neuronal excitability and epileptic activity (31). The present study indicates that gastrointestinal dysmotility in individuals with Leigh syndrome may induce malabsorption and malnutrition, which can facilitate epileptic activity.

Others have also reported a correlation between epilepsy and mitochondrial disease. For instance, Khurana et al. found that focal seizures, with or without secondary generalization, were common among those with mitochondrial disorders (12). Canafoglia et al. reported that patients with Leigh syndrome often experienced focal seizures more than they did generalized ones (4). Similarly, the present study finds an elevated proportion of focal seizure history among those with Leigh syndrome and epilepsy. Others have reported conflicting results. For instance, Lee et al. found that, of 48 patients with mitochondrial dysfunction, focal seizures were evident in 8 (16.7%), while generalized seizures were evident in 14 patients (29.2%) (13). While some results have differed from ours, the present study included various types of mitochondrial disorders, allowing for more precise resolution of participants symptomatology. Other reports of mitochondrial disorders have found slow and disorganized background rhythms with focal epileptic discharges on EEG, with over half of mitochondrial dysfunction patients exhibiting focal or multifocal spike waves and a high proportion of focal or multifocal sharp waves in patients with Leigh syndrome (4, 12). All patients assessed here exhibited slow and disorganized background EEG signs, results which agree with those reported previously. Some participants in the present study, however, presented multifocal spike wave discharges on EEG. As we have found here, Finsterer et al. also found that various subtypes of mitochondrial disorder are associated with different types of epilepsy (32). For instance, MERRF presents with generalized epilepsy and EEG spikes while MELAS presents with focal or generalized seizures and focal or multifocal EEG spikes (15, 33). Mitochondrial disorder patients assessed in the present study exhibited slow and disorganized background EEG signals, though they also exhibited multifocal or focal spikes depending on the mitochondrial disorder subtype. Other aspects of epilepsy also differ between mitochondrial disease cases. For instance, Lee et al. compared reductions in seizure frequency with more than 1 year of anti-epileptic treatment and found that 13% of syndromic mitochondrial disease patients experienced 50–75% decreases while 26% of nonsyndromic mitochondrial patients experienced decreases of <25% (34). By comparison, the results of the present study reveal that 42.8% of Leigh syndrome patients were seizure free after 1 year and that 57.1% of patients were seizure free upon their last clinic visit. These results indicate that prevalence of seizure semiology on EEG and the effects of anti-epileptic treatment may differ depending on the particular subtype of mitochondrial disorder (35).

While our data reveal some interesting trends with regard to Leigh syndrome with mtDNA mutations, the study's sample size was not large enough to parse particular subtype differences, posing a significant limitation to the present study. In this study, we investigated the association regarding epilepsy in patients with mtDNA associated Leigh syndrome only. Nevertheless, a study of epilepsy in Leigh syndrome with nuclear DNA (nDNA) mutations is further needed. A comparative study of the epilepsy pattern between Leigh syndrome with mtDNA mutation and with nDNA mutation would be worthwhile for the better understanding of Leigh syndrome.

## Conclusion

Epilepsy is a significant neurologic manifestation of Leigh syndrome. In the present study, we found an increase in the prevalence of gastrointestinal symptoms among those with Leigh syndrome and epilepsy (vs. without epilepsy), potentially due to the high energy demands associated with epilepsy. We also found that acute imaging changes may indicate an increased risk for epilepsy in individuals with Leigh syndrome, suggesting that imaging should be conducted within 2 years of diagnosis to determine the syndrome's natural course. Additionally, various types of epilepsy and differences in seizure semiology on EEG findings were also found to be associated with different mitochondrial disorders. The optimal and precise diagnosis of these disorders may lead to the increased accuracy of their diagnosis and improved treatments for epilepsy in mitochondrial disorders. Further studies that include the various subtypes of mitochondrial disorders and the use of larger samples are needed to develop improved diagnosis and treatment strategies. Given our findings, we contend that specialists in mitochondrial disorders should create individualized treatment plans and assess the disorder's natural course and prognosis in each case that they treat. As the science on these disorders moves forward, so too will its treatment.

## Ethics Statement

This study was carried out in accordance with the recommendations of the Institutional Review Board of Gangnam Severance Hospital, Yonsei University College of Medicine with written informed consent from all subjects. All subjects gave written informed consent in accordance with the Declaration of Helsinki. The protocol was approved by the Institutional Review Board of Gangnam Severance Hospital, Yonsei University College of Medicine.

## Author Contributions

Y-ML conceptualized and designed the study, coordinated and supervised data collection, and critically reviewed and revised the manuscript. SL and J-HN designed the data collection instruments, collected data, carried out the initial analyses, drafted the initial manuscript and revised the manuscript. All authors approved the final manuscript as submitted and agree to be accountable for the content of the work.

### Conflict of Interest Statement

The authors declare that the research was conducted in the absence of any commercial or financial relationships that could be construed as a potential conflict of interest.
